# Improved Mechanical and Moisture-Resistant Properties of Woven Hybrid Epoxy Composites by Graphene Nanoplatelets (GNP)

**DOI:** 10.3390/ma12081249

**Published:** 2019-04-16

**Authors:** Jesuarockiam Naveen, Mohammad Jawaid, Edi Syams Zainudin, Mohamed Thariq Hameed Sultan, Ridwan Yahaya

**Affiliations:** 1 Department of Mechanical and Manufacturing Engineering, Faculty of Engineering, Universiti Putra Malaysia, Serdang 43400, Selangor, Malaysia; gandhi.naveen66@gmail.com; 2 Laboratory of Bio composite Technology, Institute of Tropical Forestry and Forest Products (INTROP), Universiti Putra Malaysia, Serdang 43400, Selangor, Malaysia; thariq@upm.edu.my; 3 Department of Aerospace Engineering, Faculty of Engineering, Universiti Putra Malaysia, 43400 Serdang, Selangor, Malaysia; 4 Science and Technology Research Institute for Defence, Kajang 43000, Selangor, Malaysia; adr266@gmail.com

**Keywords:** kevlar, cocos nucifera sheath, graphene nanoplatelets, hybrid composites, mechanical properties, moisture diffusion, ANOVA

## Abstract

This research investigated the effect of adding different wt.% (0, 0.25, 0.50, and 0.75) of GNP (graphene nanoplatelets) to improve the mechanical and moisture resistant properties of Kevlar (K)/cocos nucifera sheath (CS)/epoxy hybrid composites. The laminates were fabricated with different K/CS weight ratios such as 100/0 (S1), 75/25 (S2), 50/50 (S3), 25/75 (S4), and 0/100 (S5). The results revealed that the addition of GNP improved the tensile, flexural, and impact properties of laminated composites. However, the optimal wt.% of GNP varies with different laminates. A moisture diffusion analysis showed that the laminates with a 0.25 wt.% of GNP content efficiently hindered water uptake by closing all the unoccupied pores inside the laminate. Morphological investigations (SEM and FE-SEM (Field Emission Scanning Electron Microscope)) proved that the addition of GNP improved the interfacial adhesion and dispersion. Structural (XRD and FTIR) analyses reveals that at 0.25 wt.% of GNP, all the hybrid composites showed a better crystallinity index and the functional groups presents in the GNP can form strong interactions with the fibers and matrix. A statistical analysis was performed using One-way ANOVA, and it corroborates that the mechanical properties of different laminates showed a statistically significant difference. Hence, these GNP-modified epoxy hybrid composites can be efficiently utilized in load-bearing structures.

## 1. Introduction

A growing awareness towards eco-friendly materials, environmental regulations, and high rates of the depletion of fossil fuels have encouraged researchers to find an alternate natural fiber to replace synthetic fiber in polymeric composites and to be compatible with the environment [1]. Recent studies have reported that natural fibers can efficiently replace the man-made synthetic fibers [2]. The main advantages of natural fibers are having a low cost, having a low density, being nonabrasive, being noncorrosive, having an inherent biodegradability, having acceptable specific strength and stiffness, being easily available, and being recyclable [3]. Reddy et al. [4] investigated the physical and mechanical properties of cocos nucifera sheath and reported that these “Natural Textiles” can be a potential and sustainable reinforcement in the polymeric composites. Moreover, Jawaid et al. [5] evaluated the influence of fiber orientation on the mechanical behaviour of natural fiber-based polymeric composites and found that laminated composites with a woven orientation provide superior mechanical properties than short fiber-based polymeric composites. However, in order to achieve a woven orientation, most of the plant fibers underwent a weaving process, which further increases the overall production cost. On the other hand, a weaving process was not required for naturally woven cocos nucifera sheath. Despite its merits, major limitations of using plant fibers are having a hydrophilic nature, having poor interfacial interactions, and having a low thermal stability with adjacent counterparts [6,7].

Hybridizing natural and synthetic fibers together with nanofillers will overcome the limitations of natural fibers for advanced applications [8,9]. Man-made synthetic fibers and natural fibers can be embedded into a single polymeric matrix to produce hybrid composites that takes advantage of individual constituents. The notion of hybridizing synthetic and natural fiber is to reduce the usage of synthetic fibers in polymeric composites. Siva et al. [10] investigated the tensile properties of Cocos nucifera sheath/glass hybrid polyester composites. They found that hybrid composites exhibited better tensile properties than glass fiber/polyester composites. Kumar et al. [11] developed and investigated the mechanical properties of cocos nucifera sheath/banana hybrid polyester composites and concluded that hybridization improved the mechanical properties compared to Cocos nucifera sheath/polyester and banana/polyester composites. Rajini et al. [12] investigated the mechanical properties of hybrid glass/cocos nucifera sheath/polyester composites, and they have proven that cocos nucifera sheaths can be a potential alternative to glass fiber in polymeric composites.

Aramid fabrics are high performance man-made fabrics with rigid polymeric chains. The molecules are connected by a strong hydrogen bond which efficiently transfers the mechanical stress. These high-performance fabrics have been widely utilized in aerospace structures and ballistic applications such as personal body armour, vehicle spall liners, etc. [13,14,15]. Yahaya et al. [2] investigated the influence of a layering pattern on the mechanical properties of Kevlar/kenaf hybrid epoxy composites and found that the tensile and flexural properties were higher for the laminates with Kevlar as an outer layer, whereas the impact strength was higher for the laminates with kenaf skin layers. Jambari et al. [16] analyzed the effect of hybridizing woven Kevlar- and kenaf-based epoxy composites and concluded that the hybrid composite showed better properties than the kenaf/epoxy composites. Yahaya et al. [17] evaluated the effect of fiber orientation on the mechanical properties of Kevlar/kenaf/epoxy composites for vehicle spall liner applications. They concluded that the tensile and impact properties were higher for woven composites than for unidirectional hybrid composites. On the contrary, longitudinally oriented hybrid composites showed better flexural properties than hybrid woven composites.

Generally, textile composites possess a higher crack propagation resistance, since fibers aligned in “warp” and “weft” directions produce a greater strength in resisting the crack opening in the polymeric matrix. Also, textile composites exhibited an improved mechanical interlocking between fibers and showed a significant reduction in anisotropy [18].

It is imperative to find sustainable materials with higher mechanical and multifunctional properties to achieve the specific requirements for particular applications. Nanofiller-modified polymeric resin was found to be an efficient, economic, and most convenient way to enhance the mechanical properties of polymeric composites [19]. The most commonly used nanofillers are carbon nano tube (CNT), nanoclay, graphene, and carbon nanofibers [20]. Graphene, one of the strongest and stiffest materials available today, possesses a greater strength (130 GPa) and modulus (1 TPa) [21]. Graphene nanoplatelets (GNPs) have become an emerging and potential nanofiller in the polymer matrix with an excellent balance between cost and properties [22]. GNPs were derived from naturally available graphite sources. Moreover, GNPs could be produced in a large scale at low cost [23].

Many researchers have devoted their efforts to produce multifunctional composites using graphene nanoplatelets as novel nanofillers for a polymeric matrix. Rafiee et al. [24] investigated the effect of adding different wt.% of GNP in the epoxy and found that the incorporation of 0.125 wt.% of GNP improved the tensile strength by 45%. Singh et al. [25] investigated the impact of adding GNP and CNT on the mechanical properties of epoxy nano composites and concluded that GNP/epoxy composites showed better mechanical properties than CNT/epoxy composites. Kamar et al. [26] analyzed the influence of adding GNP on the flexural behaviour of glass/epoxy composites and concluded that the addition of GNP (0.25 wt.%) improved the flexural strength by 29%.

The present research work investigated the effect of adding different wt.% of GNP (0, 0.25, 0.50, and 0.75) on the mechanical properties such as the tensile, flexural, and impact, moisture diffusion, morphological (SEM and FE-SEM), and structural (FTIR and XRD) properties of Kevlar-, hybrid Kevlar/cocos nucifera sheath- and cocos nucifera sheath-based epoxy composites. We also carried out statistical analyses of the obtained results by using one-way ANOVA to find the significant differences between the mean mechanical properties of different laminates.

## 2. Materials and Methods

### 2.1. Materials

**Figure 1:**
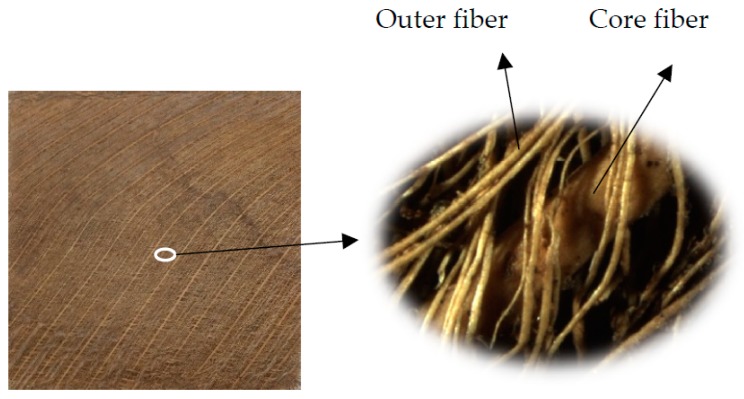
The cocos nucifera sheath [27].

Cocos nucifera sheath, a “Natural textile” and an “Agro waste”, was used in this study. The sheaths were collected from Serikembangan, Malaysia. The density of the sheath is 1.37–1.50 g/cm^3^.The properties of cocos nucifera sheaths are shown in Table 1. Figure 1 shows the collected cocos nucifera sheaths from a coconut tree. The sheath consists of core fibers which are sandwiched between two layers of outer fibers. The cocos nucifera sheaths were immersed in water for 1 week. Then the sheaths were cleaned using tap and distilled water. Eventually, the cocos nucifera sheaths were dried in the hot sun.

**Figure 2:**
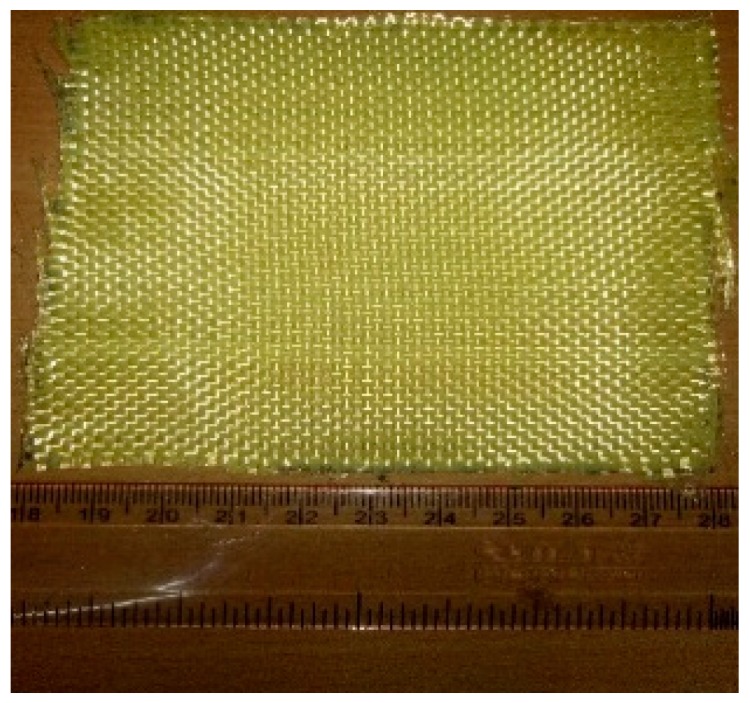
The Kevlar fabric.

**Figure 3:**
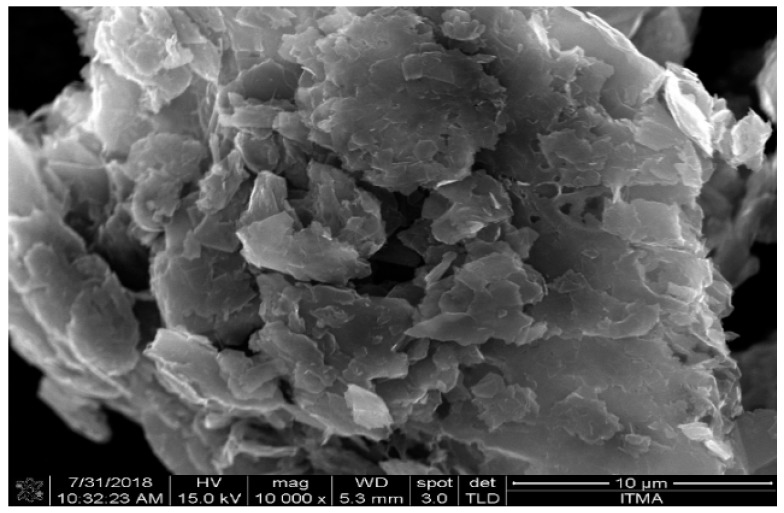
An Field Emission Scanning Electron Microscope (FE-SEM) image of the Graphene nanoplatelets (GNPs).

Figure 2 displays the Kevlar 29 fabric. The thickness and density of the fabric are 0.33 mm and 1.44 g/cm^3^, respectively. The matrix utilized in this research was liquid epoxy resin (D.E.R.331) with a density of 1.08 g/cm^3^. A joint amine (905-3S) was used as a curing agent. The GNPs were purchased from Sigma Aldrich, USA in powder form. The specifications of the GNP are listed in Table 2. Figure 3 displays the FE-SEM image of the GNPs.

### 2.2. Dispersion of GNPs in the Epoxy Matrix and Fabrication of Laminated Composites

**Figure 4:**
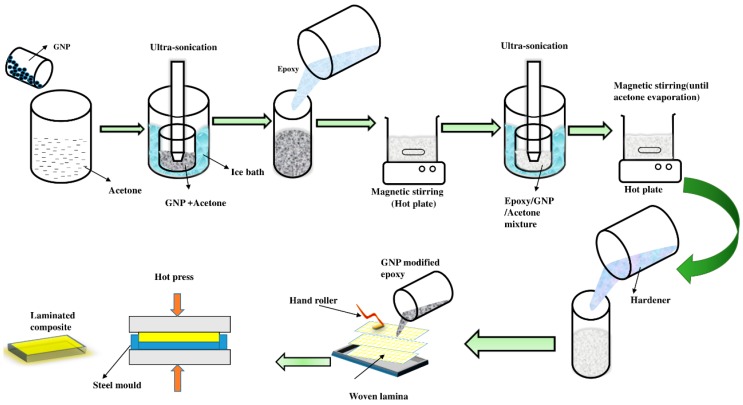
The GNP dispersion into the epoxy matrix.

Figure 4 presents the process of mixing GNPs with epoxy resin. In order to remove the residues left during the production process of GNP, they were heat-treated in a muffle furnace for 2 h at 400 °C. Then, the required amounts of GNPs (0.25 wt.%, 0.50 wt.% and 0.75 wt.%) were dispersed in acetone (GNP/acetone concentration:15 mg/ml) through an ultra-sonicator for 2 h at 90 W. The GNP/Acetone mixture was mixed with epoxy resin using a magnetic stirrer over a hot plate until the epoxy resin was completely dissolved. Further, to achieve a better dispersion, the epoxy/GNP/acetone mixture was sonicated for 30 min at 100 W. The acetone solution evaporated when the mixture was kept over a hotplate at 60 °C for 20 min under magnetic stirring. It was necessary to cool the mixture before adding the hardener. The mixture was then cooled at room temperature for 1 h. Eventually, the amine hardener was added to the epoxy/GNP mixture. The ratio of epoxy to hardener was 2:1.

The laminates were produced using a simple hand layup technique followed by hot pressing. The composite samples were made using a stainless steel mould (150 × 150 × 3 mm^3^). Silicone spray which was used as a releasing agent prevents the adhesion of the laminate to the mould. The weight ratio of the fiber and matrix was kept as 45:55. The different weight ratios of Kevlar and the cocos nucifera sheath are as follows: 100/0 (S1), 75/25 (S2), 50/50 (S3), 25/75 (S4), and 0/100 (S5). Table 3 shows the specification, layering sequence, and GNP wt.% of the different laminates. The woven mats of Kevlar and Cocos nucifera sheaths were placed inside the stainless steel mould according to the layering pattern and wetted with Epoxy/GNP mixture. Air bubbles, which may present inside the laminates, were removed using a hand roller. Eventually, the stainless steel mould was placed in a hot press for 1 h (temperature: 105 °C, Pressure: 275 bar) followed by a cold press for 15 min (Pressure: 275 bar) to prevent warpage.

### 2.3. Characterization

#### 2.3.1. Tensile Testing

The tensile properties of different laminates were examined using an INSTRON 5566 Universal Testing machine (UTM, Instron, Norwood, MA, USA) according to ASTM D 3039 standards [28] with sample dimensions of 120 mm × 20 mm × 3 mm. A standard head displacement speed (5 mm/min) was employed during testing. Five replicates were tested under each group, and the average values were reported.

#### 2.3.2. Flexural Testing

The flexural properties of the composite samples were determined using a three-point bending test at room temperature through a INSTRON 5566 Universal Testing machine (UTM) as per ASTM D 790 standards [29] with a sample dimension of 120 mm × 20 mm × 3 mm. The cross head speed was maintained at 2 mm/min. Five replicates were examined under each category, and the average values were tabulated.

#### 2.3.3. Impact Testing

An Izod impact testing was performed to investigate the impact toughness of the laminates using an impact tester (Gotech GT-7045-MD, Gotech, Taichung city, Taiwan) according to ASTM D 256 standards [30] with a sample size of 70 × 15 × 3 mm^3^. Five identical samples were examined under each category, and the average values were reported. The absorbed energy during impact is the amount of energy required to fracture the specimen completely. The impact strength or impact toughness was calculated using the following relation.

Impact strength = Impact energy/cross sectional area (KJ/m^2^) (1)

#### 2.3.4. Moisture Diffusion Analysis

The moisture diffusion properties of different laminates were evaluated according to ASTM 570 standards [31] to investigate the kinetics of moisture absorption. Initially, the laminated composite samples were dried in the furnace (100 °C) for 24 h. Then, the composite samples were immersed in distilled water (room temperature). A four digit weighing balance was used to measure the weight of the samples periodically.

The potential of water molecules to penetrate into the fiber-reinforced polymeric composites was determined using a moisture diffusion coefficient (*D*), which is an important parameter in Fick’s model. The percentage of moisture absorption was calculated using Equation (2).

(2) $$\mathrm{Moisture}\;\mathrm{absorption}\;\left(\%\right)=\frac{w_{t}-w_{i}}{w_{i}}\times 100\%$$

where *w_t_* and *w_i_* are the weight of the composite sample at time “*t*” and the “initial weight”, respectively.

The diffusion coefficient *D* (mm^2^/s) of different laminates can be determined using Equation (3).

(3) $$\mathrm{Diffusion}\;\mathrm{coefficient}\;\left(D\right)=\pi {\left(\frac{st}{4Ms}\right)}^{2}$$

where “*s*” indicates the slope of the moisture absorption curve, “*t*” represents the initial thickness of the sample, and *M_s_* is the saturated moisture absorption (%).

#### 2.3.5. Fourier Transform Infrared Spectroscopy (FTIR)

The functional groups of the individual constituents of the laminated composites were identified using an FTIR analysis through Thermo Nicolet, (Thermo fisher scientific, Waltham, MA, USA); the model was Nicolet 6700 in the range of 4000–400 cm^−1^.

#### 2.3.6. X-ray Diffraction (XRD)

The “Crystallinity” of different laminated composites was calculated using a powdered XRD (X-ray diffraction) analysis, and the patterns were collected by using the Powder X-Ray Diffractometer (Shimadzu XRD-6000, Shimadzu, Nakagyo-ku, Japan.) with Ni-filtered Cu Kα radiation at a scanning rate of 2°/min (Scanning angle: 0°–80°). The crystallinity index (*CI*) for the different composites was calculated using Equation (4) [4].

(4) $$CI=\frac{I_{C}-I_{a}}{I_{C}}\times 100\%$$

where *I_c_* and *I_a_* indicate the intensities of the crystalline and amorphous peaks, respectively.

#### 2.3.7. Morphology: Scanning Electron Microscopy (SEM)

The morphology and microstructure of the tensile-fractured laminates were studied using a SEM (Hitachi S-3400N, Hitachi, Krefeld, Germany). In order to achieve a better visualisation, gold-coated fractured samples were used.

#### 2.3.8. Field Emission Scanning Electron Microscopes (FE-SEM)

The dispersion and aggregation of GNPs in the epoxy were examined using an Ultra High-Resolution Scanning Electron Microscope (FE-SEM) (Brand: FEI, MODEL: NOVA NANOSEM 230, UNSW, Sydney, Australia). For better visualisation, the fractured composite samples were sputter coated with gold.

#### 2.3.9. Statistical Analysis

One way ANOVA was performed with “Minitab 18” (Minitab, State College, PA, USA) to evaluate the significant differences between the mean mechanical properties of different laminates.

## 3. Results and Discussion

### 3.1. Tensile Properties

**Figure 5:**
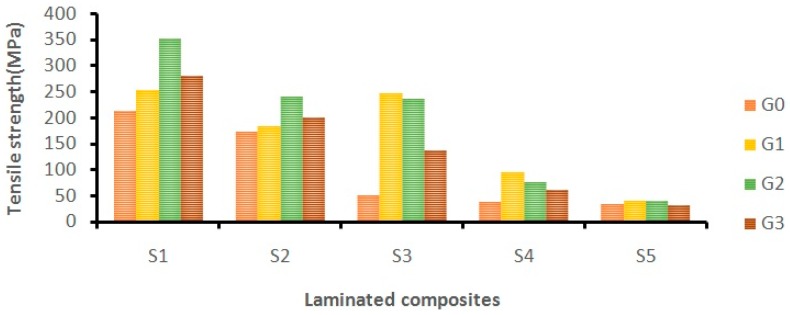
The effect of GNP on the tensile strength of laminated composites.

The tensile properties of woven fabric-reinforced polymer composites mainly depend on the weaving nature of the fabric, the layering pattern, the density, and the fiber/matrix adhesion [32]. The effect of adding different wt.% of GNP on the tensile strength of laminates is shown in Figure 5. From the analysis, it was observed that the addition of GNP improved the tensile strength of the Kevlar- (S1), hybrid- (S2, S3, and S4), and Cocos nucifera sheath (S5)-based epoxy composites. The Kevlar/epoxy (S1) composite showed improvement (63.7%) in the tensile strength up to 0.5 wt.% of GNP. At 0.75 wt.%, the tensile strength of the Kevlar/epoxy (S1) laminates declined due to GNP aggregation. Similarly, the hybrid composites (S2) and cocos nucifera sheath-based laminates (S5) showed improvements in tensile strength by 38.5% and 20.5% respectively at 0.5 wt.% of GNP. Further, the addition of GNP showed a decrement in tensile strength of S2- and S5-laminated composites. 

The improvement in tensile strength is due to the better dispersion together with the stiffening effect of rigid GNP. Also, the reinforcing effect was more prominent when the GNP wt.% was 0.5 for the S1, S2, and S5 laminates [33].

S3 and S4 hybrid composites improved the tensile strength at 0.25 wt.% of GNP by 386% and 140% respectively. The GNP improved the mechanical interlocking through a strong interface, which efficiently transferred the load to the reinforcement [20]. However, at 0.5 wt.% and 0.75 wt.%, hybrid composites S3 and S4 exhibited decrements in tensile strength due to GNP aggregation. 

**Figure 6:**
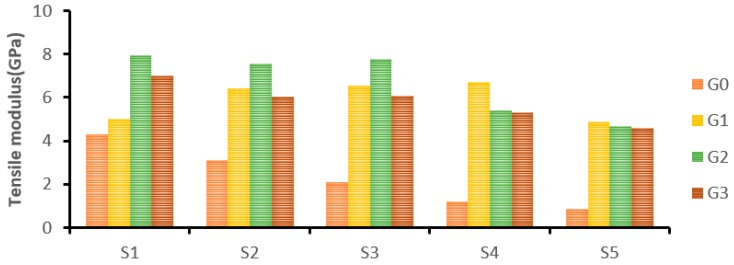
The effect of GNP on the tensile modulus of laminated composites.

Figure 6 shows the impact of adding different wt.% of GNP on the tensile modulus of different laminated composites. It was found that GNP-modified epoxy composites improved the tensile modulus of different laminates (S1, S2, S3, S4, and S5). The addition of GNP at 0.50 wt.% improved the tensile modulus of Kevlar/epoxy (S1) composites by 84.44%. Similarly, hybrid composites S2 and S3 showed improvements in tensile modulus by 143.5% and 271.42% respectively at 0.50 wt.% of GNP content. At 0.75 wt.% of GNP content, the laminated composites S1, S2, and S3 showed decrements in the tensile modulus due to GNP aggregation. Hybrid composite S4 and the cocos nucifera sheath/epoxy composites (S5) improved the tensile modulus by 450% and 460% respectively at 0.25 wt.% of GNP content. 

Generally, the cellulose content of the natural fiber is attributed to its tensile properties. The addition of GNP drastically improved the tensile properties of the laminates (S2, S3, S4, and S5), though the cellulose content of CS is very low (22.5%) [34]. The higher modulus of the laminates reveals that the structure is more rigid and stiff. Hence, a higher stress level is required to achieve the desired strain.

The addition of GNP improved the tensile properties of the laminates, and it is mainly due to the stiffening effect of forming a rigid structure together with the improved crystallinity and mechanical interlocking.

A statistical analysis was carried out using ANOVA (one-way) to find the significant difference between the mean tensile strength as well as the modulus of Kevlar-, hybrid-, and cocos nucifera sheath-reinforced GNP-modified epoxy composites. Five replicates were tested under each of the twenty laminated composites. The variance of tensile strength and modulus were divided into two categories such as BG (Between Group) and WG (Within Group). The F-Value is the ratio between the mean square of BG to the mean square of WG. The *p*-value of the F-test is less than 0.05 in Table 4 and Table 5, which reject the null hypothesis. From the ANOVA test, it was found that a statistically significant difference exists between the mean tensile strength of the laminates as well as the mean modulus of the laminates with a 95% confidence level.

### 3.2. Flexural Properties

**Figure 7:**
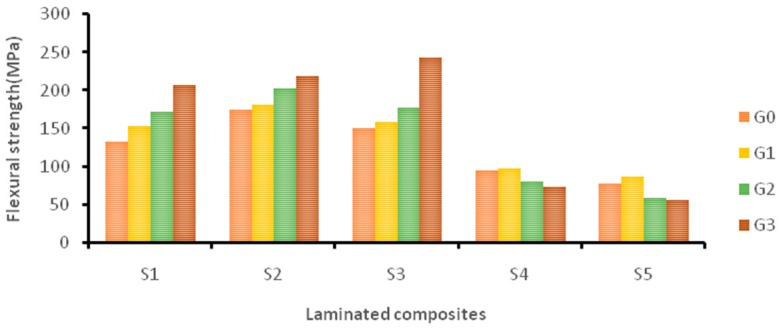
The effect of GNP on the flexural strength of laminated composites.

Figure 7 shows the flexural strength of the laminated composites at different wt.% of GNP. The addition of GNP improved the flexural strength of pure Kevlar composites (S1) by 56.16% at 0.75 wt.% of GNP content. Similarly, hybrid composites S2 and S3 showed improvements at 0.75 wt.% of GNP by 24.14% and 61.23% respectively. Due to the addition of more cocos nucifera sheaths, hybrid composite S4 showed a higher flexural strength at 0.25 wt.% of GNP by 2.2%. Similarly, pure cocos nucifera sheath composites (S5) showed an 11.91% improved flexural strength at 0.25 wt.% of GNP. However, there was no improvement observed at 0.5 and 0.75 wt.% due to GNP agglomeration.

The addition of GNP improved the flexural strength of different Kevlar- and cocos nucifera sheath-based laminates (S1 to S5). This is due to the formation of a strong interfacial bond between the reinforcement and epoxy through GNP [35,36]. Khalil et al. also reported that a strong interface between the reinforcement and matrix exhibited a higher flexural strength [15].

**Figure 8:**
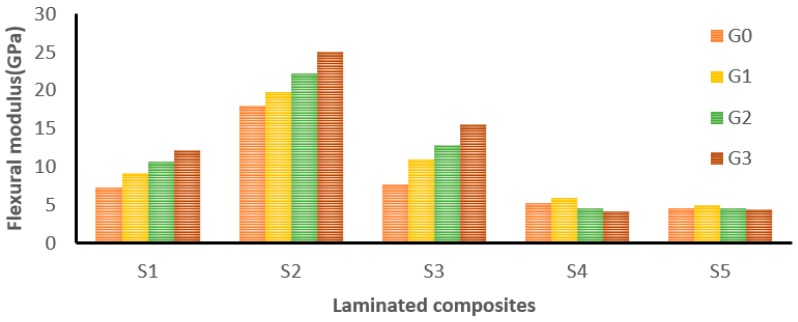
The effect of GNP on the flexural modulus of laminated composites.

Figure 8 shows the influence of adding different wt.% of GNP on the flexural modulus of laminated composites (S1 to S5). Among the laminated composites, the S2 hybrid composite showed a higher flexural modulus at 0 wt.% of GNP. It has proved that hybrid composites take the advantage of individual constituents which results in superior properties [2]. Kevlar fabric-based (S1) composites showed a 67.26% improvement in flexural modulus at 0.75 wt.% of GNP. Similarly, S2 and S3 hybrid composites exhibited 39.55% and 101.4% improvements in flexural modulus at 0.75 wt% of GNP. The S4 and S5 laminates showed slight improvements (13% and 7.6% respectively) at 0.25 wt.% of GNP. However, there was no improvement observed at 0.50 and 0.75 wt.% due to GNP agglomeration.

In this work, the optimal flexural properties were found at 0.75 wt.% of GNP for S1, S2, and S3 laminates, whereas for S4 and S5, the optimum GNP concentration was 0.25 wt.%. Kamar et al. also reported that the addition of GNP beyond 0.25 wt.% declined the flexural properties of glass/epoxy composites [26]. It can be validated with the following fact that the GNP concentration at 0.25 wt.% induces failure in the tension side or bottom layer which is due to the ability of the epoxy polymer to transfer stress to the reinforcement and rigid GNP. On the other hand, higher GNP concentrations declined the interlaminar adhesion which has led to debonding and micro-buckling on the compressive side or top layer.

A statistical analysis was carried out using ANOVA (one-way) to find the significant difference between the mean flexural strength as well as the mean modulus of different laminates. Five replicates were tested under each of the twenty laminates. The *p*-value of F-test is less than 0.05 in Table 6 and Table 7, which reject the null hypothesis. The ANOVA test results confirm that a statistically significant difference exists between the mean flexural strength of the laminates as well as the mean modulus of the laminates with a 95% confidence level.

### 3.3. Impact Toughness

**Figure 9:**
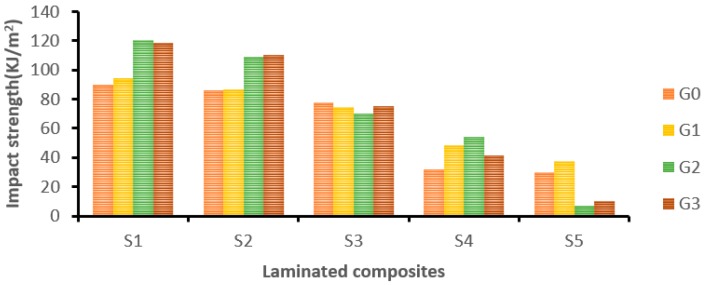
The effect of GNP on the impact strength of laminates.

Figure 9 shows the effect of adding GNP on the impact behaviour of the laminated composites (S1 to S5). At 0.50 wt.% of GNP, the S1-laminated composite showed a significant improvement in impact strength by 33.67%. The reduction in impact strength at 0.75 wt.% may be due to the formation of stress concentration in the vicinity of GNP [37]. Hybrid composite S2 showed a higher impact strength at 0.75 wt.% of GNP, and the percentage of improvement was 27.9% compared to 0 wt.% of GNP content. There was not much improvement observed in hybrid composite S3. However, hybrid composite S4 improved the impact strength by 68.8% at 0.5 wt.% of GNP content. Pure cocos nucifera sheath-based composites (S5) exhibited a higher impact strength at 0.25 wt.% of GNP by 25.86%.

It was found from Figure 9 that there was a sudden drop in the impact strength at 0.50 and 0.75 wt.% of GNP due to the excessive aggregation of GNP. Moreover, GNP agglomeration has led to defects in the matrix which act as a seed point for crack initiation and propagation [38]. Also, beyond the optimal GNP concentration, the nanoplatelets started to overlap with each other. According to Inuwa et al., a higher GNP loading has led to restacking due to strong van der Waals’ forces and π →π interactions between GNP planes and the small distance between the GNP. These are all the presumed factors responsible for the decrement in the impact strength of the laminates [39].

A statistical analysis was carried out using one-way ANOVA to find the significant difference between the mean impact strength of different laminates. Five replicates were examined under each of twenty laminates. The *p*-value of the F-test is less than 0.05 in Table 8, which rejects the null hypothesis. The ANOVA test results corroborate that a significant difference exists between the mean impact strength of the laminates with a 95% confidence level.

### 3.4. Moisture Diffusion Behaviour

**Figure 10:**
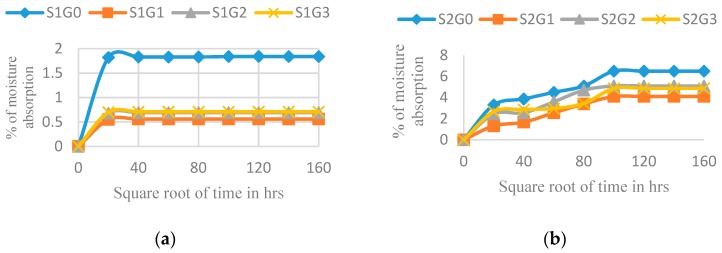

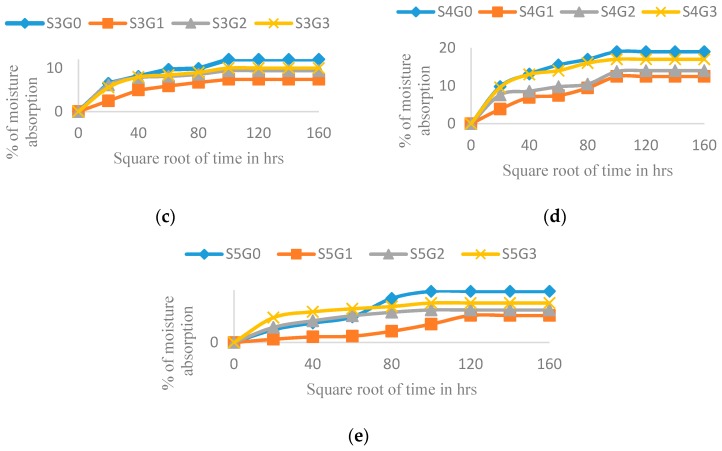
The effect of GNP on the water sorption behaviour of hybrid laminates. (**a**) S1; (**b**) S2; (**c**) S3; (**d**) S4; and (**e**) S5.

Figure 10 shows the effect of adding different wt.% of GNP on the moisture absorption of all the laminates (S1, S2, S3, S4, and S5). The addition of GNP efficiently suppresses the moisture absorption of all the laminated composites. This affirmation has been corroborated by the diffusion coefficient (Table 9). The laminates at 0.25 wt.% of GNP efficiently hinder water uptake because all the unoccupied pores inside the laminates were closed, and it shows the best moisture prevention composition. Silvia et al. also reported that the addition of a graphene nanofiller showed decreases in moisture absorption as well as in the diffusion coefficient [40]. A decrease in moisture absorption due to the addition of nanofillers can be explained by the following two effects: (i) GNP acts as an efficient barrier against moisture diffusion because of the increased tortuosity for water molecules diffusing through the epoxy matrix, and (ii) the nano-sized GNPs hinder intermolecular movement of the epoxy resin, which affects the relaxation of polymeric chain segments [41,42]. The addition of more GNP (0.50 and 0.75 wt.%) did not show much water resistance compared to the laminates with 0.25 wt.% of GNP. It is mainly because the addition of more GNPs resulted in nonuniform dispersion which leads to agglomeration as well as wrinkling. Hence, the restriction to intermolecular movement has become ineffective and enhanced moisture absorption [40]. However, the laminate with 0.50 and 0.75 wt.% of GNP exhibited better moisture resistance than the laminates with 0 wt.% of GNP.

### 3.5. FTIR

**Figure 11:**
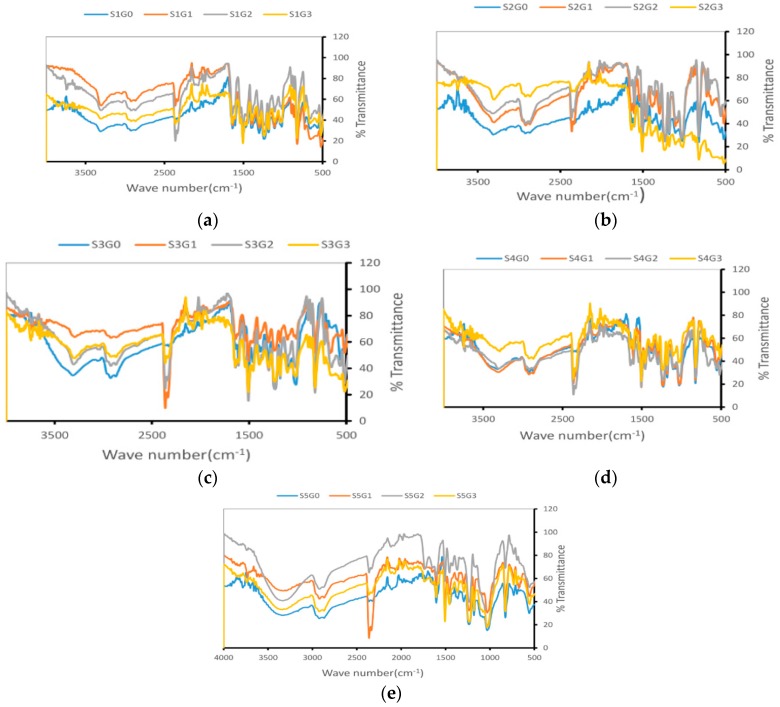
The FTIR spectra of Kevlar, Cocos nucifera sheath, and hybrid composites. (**a**) S1; (**b**) S2; (**c**) S3; (**d**) S4; and (**e**) S5.

The FTIR spectra of the laminated composites (S1 to S5) are shown in Figure 11. α-cellulose of the cocos nucifera sheath was confirmed from the bands at around 3435 cm^−1^ and 2930 cm^−1^ [4], whereas the bands at 1734 cm^−1^ and 1248 cm^−1^ corresponded to hemicellulose as shown in Figure 11b–e [43]. All the composites contain epoxy resin with an amine hardener. The bands corresponding to 1580–1650 cm^−1^ indicate the stretching of C–H of the oxirane ring and N–H bending, which clearly represents the aromatic nature of the epoxy resin. The presence of the amine hardener was confirmed with the aliphatic CH_2_ and CH_3_ broad vibrations between 3000 and 2850 cm^−1^. Also, the peaks at 915 cm^−1^, 1036 cm^−1^, 1362 cm^−1^, 1509 cm^−1^, and 1608 cm^−1^ represent the C–O of oxirane group stretching, C–O–C of ethers stretching, C–O of aldehyde stretching, C–C aromatic stretching, and C=C of aromatic ring stretching respectively [44]. The adsorption peak at 1376 cm^−1^ indicates the symmetrical bending of the CH_3_ epoxy ring [45]. The functional groups of Kevlar has been confirmed with three main peaks in the Figure 11a–d. The peak at 3300 cm^−1^ represents the –NH– stretching frequency. The peaks at 1650 cm^−1^ and 1540 cm^−1^ correspond to the stretching and bending frequency of the C=O and –NH– bonds, respectively [46]. From the FTIR spectra, it is clear that except S1G0, S2GO, S3GO, S4G0, and S5G0, all other composites that contain GNP showed a broad band between 2200–2400 cm^−1^ corresponding to CC stretching vibration and O–H and N–H stretching vibration. The FTIR analysis reveals that the functional groups present in the GNP can form strong interactions with Kevlar, Cocos nucifera sheath, and epoxy resin.

### 3.6. XRD

**Figure 12:**
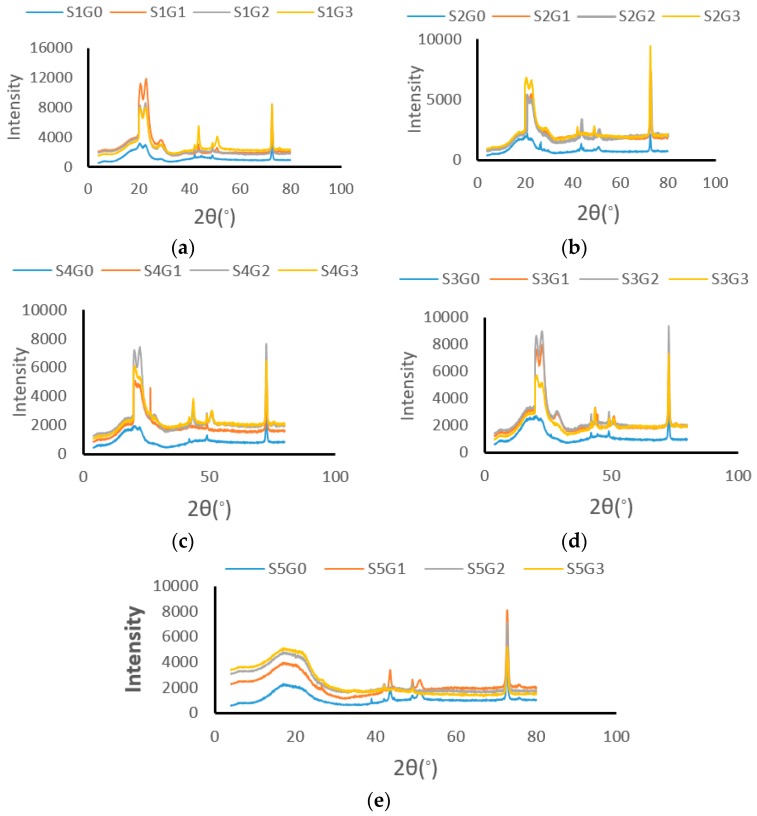
The X-Ray diffractograms of hybrid composites. (**a**) S1; (**b**) S2; (**c**) S3; (**d**) S4; and (**e**) S5.

An XRD analysis was employed to evaluate the crystalline nature of Kevlar/cocos nucifera sheath/GNP epoxy composites. Generally, the broad hump pattern in the diffractogram represents the amorphous constituents, while the sharp peak corroborates the presence of crystalline constituents in the composites. Figure 12 shows the effect of GNP on the X-ray diffraction patterns of different hybrid composites (S1, S2, S3, S4, and S5). 

The wide peak around 20° (*I_a_*) and hte sharp peak at 74° (*I_c_*) indicate the amorphous and crystalline compounds (cellulose) present in the composites, respectively. The typical patterns in Figure 12a–d at 2θ = 20.5 (110), 22.6° (200), and 29.3° (211) were attributed to Kevlar, which are in good agreement with the previous literatures, whereas these patterns were not found in Figure 12e, which confirms the absence of Kevlar in S5 composites [47]. Moreover, the broad peak at 2θ = 18° in Figure 12d was attributed to the amorphous polymer and lignin and hemicellulose present in the cocos nucifera sheath. Reddy et al. also found similar results while using cocos nucifera sheath [4]. A small peak at 2θ = 26 (200) represents the reflection of GNP [48]. 

Table 10 shows that the addition of GNP improved the CI of laminates. However, a higher GNP concentration eclined the CI due to nonuniform dispersion and agglomeration.

### 3.7. Morphological Analysis

**Figure 13:**
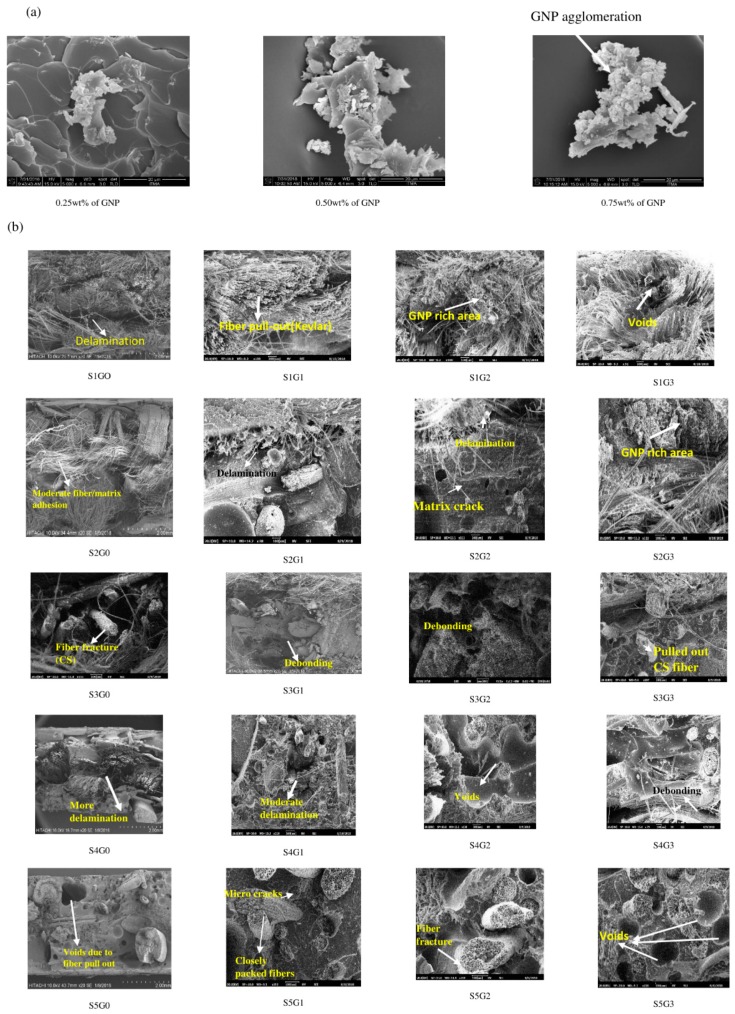
(**a**) The FE-SEM images of the tensile-fractured specimens at different GNP wt.% and (**b**) the SEM images of tensile-fractured specimens.

A fractographic investigation was carried out using SEM and FE-SEM for the tensile fractured specimens. Fiber/matrix interfacial adhesion plays an important role on the mechanical properties of the laminated composites. Generally, the reinforcement could bear the load, while the matrix transfers the load to the reinforcement. Figure 13a shows the FE-SEM fractography of the tensile fractured specimens at different GNP wt.% (0.25, 0.50, and 0.75). From the analysis, it has been found that the GNPs were agglomerated at 0.75 wt.%. Figure 13b shows the SEM fractography of the tensile fractured laminates (S1, S2, S3, S4, and S5) at different GNP wt.%. The fractography reveals that the laminates at 0.25 wt.% (S1G1, S2G1, S3G1, S4G1, and S5G1) showed improved interfacial interactions (moderate delamination) compared to the laminates at 0 wt.% of GNP (S1G0, S2G0, S3G0, S4G0, and S5G0). Excessive void contents were observed in the laminates with 0.50 wt.% and 0.75 wt.% of GNP (S1G2, S2G2, S3G2, S4G2, S5G2, S1G3, S2G3, S3G3, S4G3, and S5G3). The woven mat consists of fibers in both the warp and weft directions and interlaced with each other. Tensile loading stretches the fiber in the loading direction. However, in addition to the longitudinal stretching, the fibers arranged in the transverse direction also tend to straighten which may induce stress concentration at the interface. It forms microcracks in the polymer and propagates in the transverse direction causing fiber fracture. This continues until complete tensile failure occurs.

## 4. Conclusions

The effect of adding different wt.% of GNP on the mechanical, moisture diffusion, and morphological analyses of hybrid Kevlar/cocos nucifera sheath composites was studied. The following conclusions were drawn from the analysis.

- The addition of GNPs improved the mechanical properties such as tensile, flexural, and impact properties of different laminates (Kevlar/GNP/Epoxy, hybrid laminates, and cocos nucifera sheath/GNP/epoxy). This is mainly due to a better dispersion of the rigid GNP which enhances the mechanical interlocking capability by bridging individual plies through a strong interface. However, the optimized GNP concentration varies depending on the wt.% of the Kevlar and cocos nucifera sheaths in the hybrid laminates.
- The addition of GNP efficiently suppresses the water uptake of all the laminated composites. At 0.25 wt.% of GNP, the laminates efficiently hinder water uptake because all the unoccupied pores inside the laminates were closed. However, the laminates with 0.50 and 0.75 wt.% of GNP did not show much water resistance due to the nonuniform dispersion which leads to agglomeration as well as wrinkling.
- An FTIR analysis reveals that the functional groups present in the GNP can form strong interactions with Kevlar, Cocos nucifera sheath, and epoxy resin. From the XRD analysis, it was found that the addition of GNP improved the crystallinity of the hybrid composites. It validates the improvement in mechanical properties of hybrid laminates. However, a higher concentration of GNP slightly declined the crystallinity.
- A fractography of the tensile-fractured specimen showed that the addition of GNP improved the interfacial interactions and reduces delaminations. However, a higher GNP concentration exhibited agglomeration, voids, and debonding due to GNP overlapping and wrinkling.

From the overall analysis, it has been concluded that these GNP-modified epoxy hybrid composites can be effectively used in loadbearing structures in the automotive, aircraft, and defence sectors where a higher strength to weight ratio is a prime requirement.

## Figures and Tables

**Table 1:** The properties of the core and outer fiber of the cocos nucifera sheath [27].

Properties		Core Fiber	Outer Fiber
Chemical Composition	Cellulose	22.25	21.99
	Hemicellulose (%)	42.01	43.44
	Lignin (%)	33.32	31.98
	Extractive (%)	2.05	2.42
	Others (%)	0.37	0.17
Physical and Mechanical Properties	Diameter (μm)	2111.6	308.08
	Tensile Strength (MPa)	169.64	69.67
	Tensile Modulus (GPa)	5.7	3.3
	% of Elongation	15.5	21.32

**Table 2:** The specifications of GNPs.

Lateral Dimension	Bulk Density	Surface Area
2–3 μ	0.2 g/mL	110 m^2^/g

**Table 3:** The specification of the laminates.

Sl. No	Symbol	No of Layers		Layering Pattern	GNP (wt.%)
		Kevlar	Cocos Nucifera Sheath		
1	S1G0	4	0	K/K/K/K	0
2	S1G1				0.25
3	S1G2				0.50
4	S1G3				0.75
5	S2G0	3	1	K/CS/K/K	0
6	S2G1				0.25
7	S2G2				0.50
8	S2G3				0.75
9	S3G0	2	2	K/CS/CS/K	0
10	S3G1				0.25
11	S3G2				0.50
12	S3G3				0.75
13	S4G0	1	3	CS/CS/K/CS	0
14	S4G1				0.25
15	S4G2				0.50
16	S4G3				0.75
17	S5G0	0	4	CS/CS/CS/CS	0
18	S5G1				0.25
19	S5G2				0.50
20	S5G3				0.75

K, Kevlar; CS, Cocos nucifera sheath.

**Table 4:** The ANOVA test results of tensile strength.

Source	DF	SS	MS	F-Value	*p*-Value
Between Group (BG)	19	960008	50526.7	9697.19	0.000
Within group (WG)	80	417	5.2		

DF, Degrees of freedom; SS, Sum of Square; MS, Mean Square.

**Table 5:** The ANOVA test results of tensile modulus.

Source	DF	SS	MS	F-Value	*p*-Value
Between Group (BG)	19	397.715	20.9324	4897.03	0.000
Within group (WG)	80	0.342	0.0043		

DF, Degrees of freedom; SS, Sum of Square; MS, Mean Square.

**Table 6:** The ANOVA test results of flexural strength.

Source	DF	SS	MS	F-Value	*p*-Value
Between Group (BG)	19	316556	16660.9	4814.68	0.000
Within group (WG)	80	277	3.5		

DF, Degrees of freedom; SS, Sum of Square; MS, Mean Square.

**Table 7:** The ANOVA test results of flexural modulus.

Source	DF	SS	MS	F-Value	*p*-Value
Between Group (BG)	19	4028.96	212.050	6625.43	0.000
Within group (WG)	80	2.56	0.032		

DF, Degrees of freedom; SS, Sum of Square; MS, Mean Square.

**Table 8:** The test results of impact strength.

Source	DF	SS	MS	F-Value	*p*-Value
Between Group (BG)	19	1146	6049.79	5152.2	0.000
Within group (WG)	80	94	1.17		

DF, Degrees of freedom; SS, Sum of Square; MS, Mean Square.

**Table 9:** The moisture diffusion analysis.

Composite Symbol	GNP (wt.%)	Percentages of Water Uptake at Saturation Time Q_S_ (%)	Diffusion Coefficient *D* (×10^−5^ mm^2^/s)
S1G0	0.00	1.84	0.014
S1G1	0.25	0.56	0.007
S1G2	0.50	0.69	0.008
S1G3	0.75	0.71	0.009
S2G0	0.00	6.51	1.92
S2G1	0.25	4.10	0.96
S2G2	0.50	5.10	1.34
S2G3	0.75	4.87	1.23
S3G0	0.00	12.12	2.04
S3G1	0.25	7.43	1.02
S3G2	0.50	9.41	1.05
S3G3	0.75	10.11	1.06
S4G0	0.00	19.13	4.89
S4G1	0.25	12.45	2.7
S4G2	0.50	14.21	3.1
S4G3	0.75	17.12	3.3
S5G0	0.00	44.12	8.25
S5G1	0.25	23.22	3.04
S5G2	0.50	28.12	3.93
S5G3	0.75	34.31	4.97

**Table 10:** The crystallinity index of different laminates.

GnP (wt.%)	Crystallinity Index (%)				
	S1	S2	S3	S4	S5
G0 (0 wt.%)	59.20	48.57	65.60	64.01	59.53
G1 (0.25 wt.%)	62.51	65.18	66.25	67.72	62.93
G2 (0.50 wt.%)	61.10	65.55	65.27	66.91	48.52
G3 (0.75 wt.%)	60.66	65.60	58.90	53.82	31.41
